# Detection and Classification of Measurement Errors in Bioimpedance Spectroscopy

**DOI:** 10.1371/journal.pone.0156522

**Published:** 2016-06-30

**Authors:** David Ayllón, Roberto Gil-Pita, Fernando Seoane

**Affiliations:** 1 R&D Department, Fonetic, 28037 Madrid, Spain; 2 Signal Theory and Communications Department, University of Alcala, Alcalá de Henares, Spain; 3 Faculty of Care Science, Work Life and Social Welfare, University of Boras, Boras, Sweden; 4 School of Technology and Health, Royal Institute of Technology, Huddinge, Sweden; US Naval Reseach Laboratory, UNITED STATES

## Abstract

Bioimpedance spectroscopy (BIS) measurement errors may be caused by parasitic stray capacitance, impedance mismatch, cross-talking or their very likely combination. An accurate detection and identification is of extreme importance for further analysis because in some cases and for some applications, certain measurement artifacts can be corrected, minimized or even avoided. In this paper we present a robust method to detect the presence of measurement artifacts and identify what kind of measurement error is present in BIS measurements. The method is based on supervised machine learning and uses a novel set of generalist features for measurement characterization in different immittance planes. Experimental validation has been carried out using a database of complex spectra BIS measurements obtained from different BIS applications and containing six different types of errors, as well as error-free measurements. The method obtained a low classification error (0.33%) and has shown good generalization. Since both the features and the classification schema are relatively simple, the implementation of this pre-processing task in the current hardware of bioimpedance spectrometers is possible.

## 1 Introduction

Electrical Bioimpedance (EBI) is a mature and well spread technological application within several clinical fields like nutrition [1, 2], renal failure [3], skin cancer screening for melanoma [4], and many others being under development like brain monitoring [5, 6], limb edema detection in children [7], regional ventilation and perfusion monitoring [8] or assessment of volume status in patients before and after general anaesthesia [9]. Bioimpedance Spectroscopy (BIS) is one of the modalities of EBI that has enabled such spread of uses. Given the intrinsic requirement of BIS to analyze bioimpedance information at several frequencies within a given frequency range, it is required that the bioimpedance spectrometer used to obtain the bioimpedance recordings produces high accurate and robust bioimpedance spectra at all frequencies.

When measuring BIS, the measurement scenario can suffer from many different sources of disturbances [10] producing measurement errors and artifacts. The most common is the influence of capacitive leakage in the estimation of the measured impedance [11–13], but very often it is possible to obtain BIS measurements with artifacts produced by other kind of errors [14, 15] or their combination [16]. A successful BIS analysis requires a measurement as clean and reliable as possible, ready for analysis. With methods and techniques for correcting [13], compensating [11, 12, 17] or avoiding [18] certain measurement errors or their influence in their analysis [19–21], the steps missing in the measurement pre-processing chain is first to detect the clean BIS measurements from those containing artifacts and second (if necessary) to identify the kind or error present in the measurement.

In this paper we present a signal pre-processing method to identify those BIS measurements clean and ready for further data analysis and those measurements that contain common measurement artifacts. A supervised machine learning approach is used to detect and classify BIS measurement errors using generalist features for measurement characterization in different immittance planes. With the objective of generalization, a simple classification scheme based on linear discriminants and a feature selection algorithm based on evolutionary computation is designed for dimensionality reduction.

### 1.1 Cole Equation

In 1940, Cole [22] introduced a mathematical equation that fitted experimentally obtained BIS measurements Eq (1). This equation is not only commonly used to represent but also to model [23] and analyze the BIS data [24]. The analysis is based on the four parameters contained in the Cole equation *R*_0_, *R*_∞_, *α*, and *τ*, which is the inverse of the characteristic frequency *ω*_*c*_.

$$\begin{array}{c} Z\left(\omega \right)=R_{\infty }+\frac{R_{0}-R_{\infty }}{1+{\left(j\omega \tau \right)}^{\alpha }}. \end{array}$$ (1)

**Fig 1:**
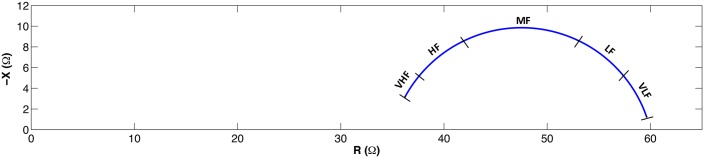
Cole plot and frequency bands definition.

The value generated by the Cole equation is a complex value, the impedance, with a non-linear relationship with the frequency that generates a suppressed semi-circle in the impedance plane. An example of a Cole function is plotted in Fig 1.

Five frequency bands can be defined as a function of the characteristic frequency *ω*_*c*_: VLF (*ω* < *ω*_*c*_/5), LF (*ω*_*c*_/5 ≤ *ω* < *ω*_*c*_/2), MF (*ω*_*c*_/2 ≤ *ω* < 2*ω*_*c*_), HF (2*ω*_*c*_ ≤ *ω* ≤ 5*ω*_*c*_), VHF (*ω* > 5*ω*_*c*_). Fig 1 also represents the margins of these frequency bands over the Cole function.

## 2 Types of BIS measurement errors

**Fig 2:**
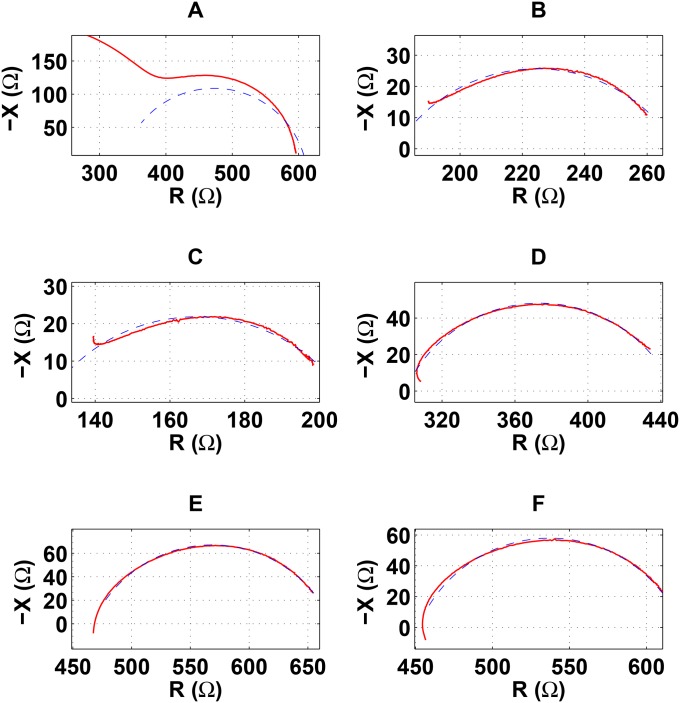
BIS measurements (solid red line) containing the 6 identified types of measurement errors plotted in the impedance plane. Estimations of the clean measurements are represented by dashed blue lines. Type-A error (A), Type-B error (B), Type-C error (C), Type-D error (D), Type-E error (E), Type-F error (F).

In order to identify the different types of BIS measurement errors, we have visually analyzed a large number of BIS measurements from different applications, identifying 6 different types of errors [11–16]. Fig 2 represents BIS measurements (solid red line) plotted in the impedance plane containing the identified types of measurement errors. Estimations of the clean measurements are represented by dashed blue lines. From the measurement artifact produced in the impedance plot, we can differentiate two groups of errors. The errors of the first group (Type-A, Type-B and Type-C) are characterized by an increment of the capacitance at the higher frequencies, usually associated to the presence of parasitic capacitive leakage. However, the capacitance of the errors in the second group (Type-D, Type-E and Type-F) always decreases with frequency beyond the characteristic frequency, as in any error-free BIS measurement, but in an excessive manner crossing the resistance axis at higher frequencies, thus changing the sign of the reactance from negative to positive, i.e. changing from capacitance to inductance.

The Type-A error is the most common BIS measurement error and it is commonly named Hook effect [11–13]. It is characterized by an early decrement of the reactance starting in the MF or HF bands. Type-B and Type-C errors also contain capacitive effects but the reactance only decreases in the highest frequencies, which originates an outer tail in the Cole function. In Type-B error the capacitive effect is only noticeable in the VHF band but in Type-C error it is already noticeable from the HF band.

Regarding the second group, Type-D error is characterized by a resistance decrement in the higher frequencies (the normal situation is that resistance always increases with frequency), which originates an inner tail at the end of the Cole function. This effect is probably accompanied by an excessive decrement in the reactance of the VHF band. In Type-E and Type-F errors, an abnormal increment in the reactance in the VHF band causes that the reactance gets positive. In Type-E error the resistance keeps decreasing with frequency. However, in Type-F error there is a resistance increase in the VHF band that also causes an inner tail similar than in Type-D error (but with positive reactance values).

## 3 Proposed Algorithm for Detection and Classification of BIS measurement errors

In order to detect BIS measurements with errors and classify the error in one of the six aforementioned types, we propose to design a supervised machine learning system trained with labeled real measurements. The detection and classification problem is solved by classifying BIS measurements in one of the next seven classes: one class corresponds with measurements without errors and the other six classes for each of the different types of measurement errors. A set of linear classifiers are designed using features based on relative errors between the BIS measurement and its estimated value obtained through Cole model fitting. Since we are considering relative errors within frequency ranges defined accordingly to the characteristic frequency, the features are valid for any BIS application, regardless of its typical Cole parameter values. With the aim of reducing both the possibility of overfitting of the learning algorithm and its computational cost, linear classification is used and a feature selection process is carried out. In addition, the classification problem is tackled with two approaches: ‘all-at-once’ approach where the seven classes are classified at the same time, and ‘divide and conquer’ approach where some classes are merged together and classified in a first step, and then the subclasses that merged into a class are classified in a second step.

### 3.1 Spectral Immittance components

Immittance englobes both impedance and admittance domains. Both impedance and admittance, denoted by *Z*(*ω*) and *Y*(*ω*) respectively, are spectral functions related to each other according to *Z*(*ω*)/*Y*(*ω*) = 1. Both are also built up by two differentiated components and require the use of complex notation to represent them. Resistance (*R*(*ω*)) and reactance (*X*(*ω*)) are the components for the impedance, and conductance (*G*(*ω*)) and susceptance (*B*(*ω*)) are the components for the admittance. Notation and relationships are indicated in Table 1.

**Table 1:** Relationship between spectral immittance components.

	*R*(*ω*)	*X*(*ω*)	*G*(*ω*)	*B*(*ω*)
*Z*(*ω*)	*Real*{*Z*(*ω*)}	*Imag*{*Z*(*ω*)}	$\frac{R(\omega )}{R{\left(\omega \right)}^{2}+X{\left(\omega \right)}^{2}}$	$\frac{-X(\omega )}{R{\left(\omega \right)}^{2}+X{\left(\omega \right)}^{2}}$
*Y*(*ω*)	$\frac{G(\omega )}{G{\left(\omega \right)}^{2}+B{\left(\omega \right)}^{2}}$	$\frac{-B(\omega )}{G{\left(\omega \right)}^{2}+B{\left(\omega \right)}^{2}}$	*Real*{*Y*(*ω*)}	*Imag*{*Y*(*ω*)}

**Fig 3:**
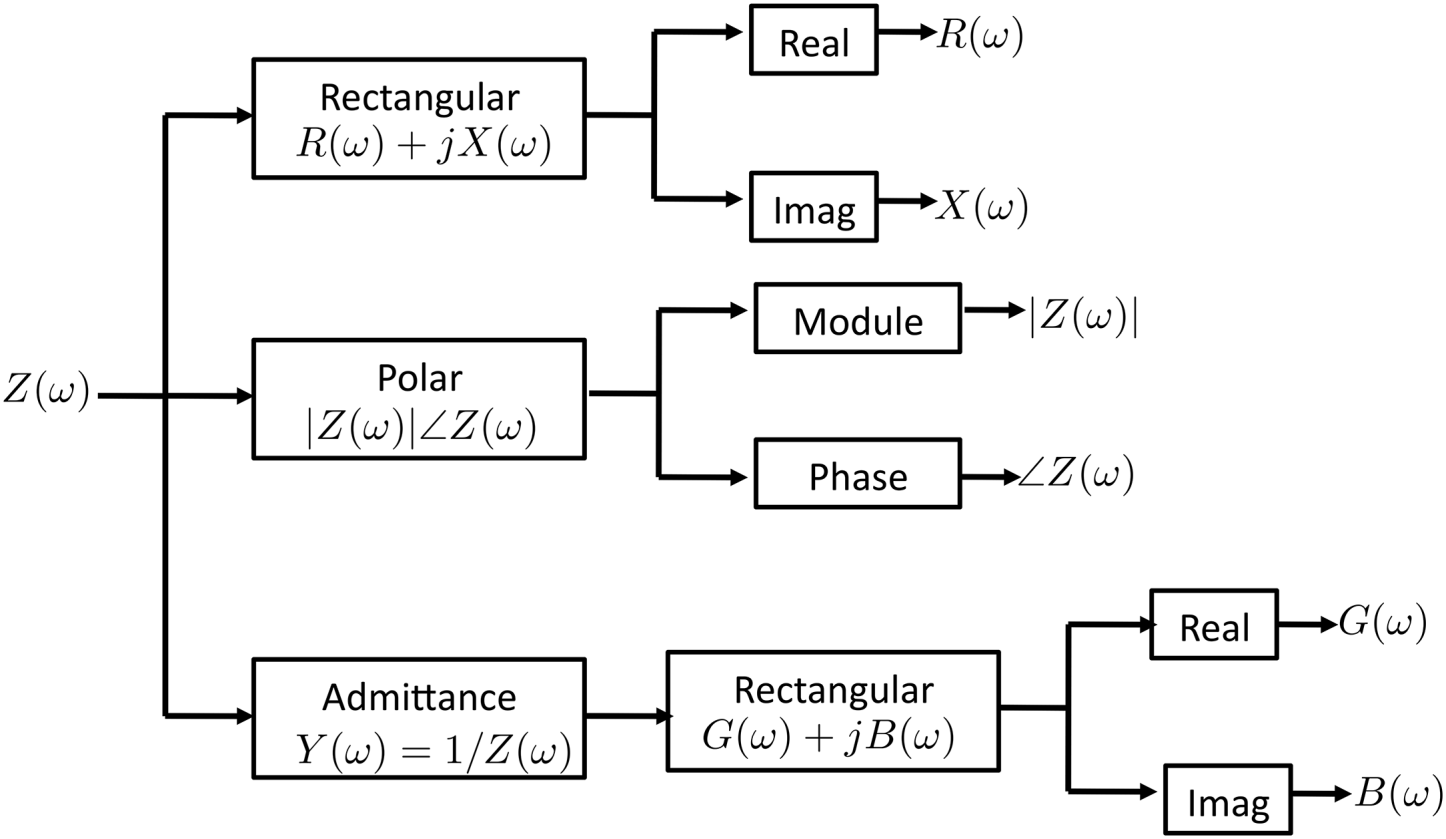
Immittance components computation.

Therefore, by measuring complex impedance or complex admittance any of the other immittance components can be calculated by computing the inverse magnitude and extracting real and imaginary parts. While the Cole function was defined originally on terms of complex impedance, it is very straight forward to calculate its admittance version and extract its real, imaginary, modulus or phase components, as shown in Fig 3.

**Fig 4:**
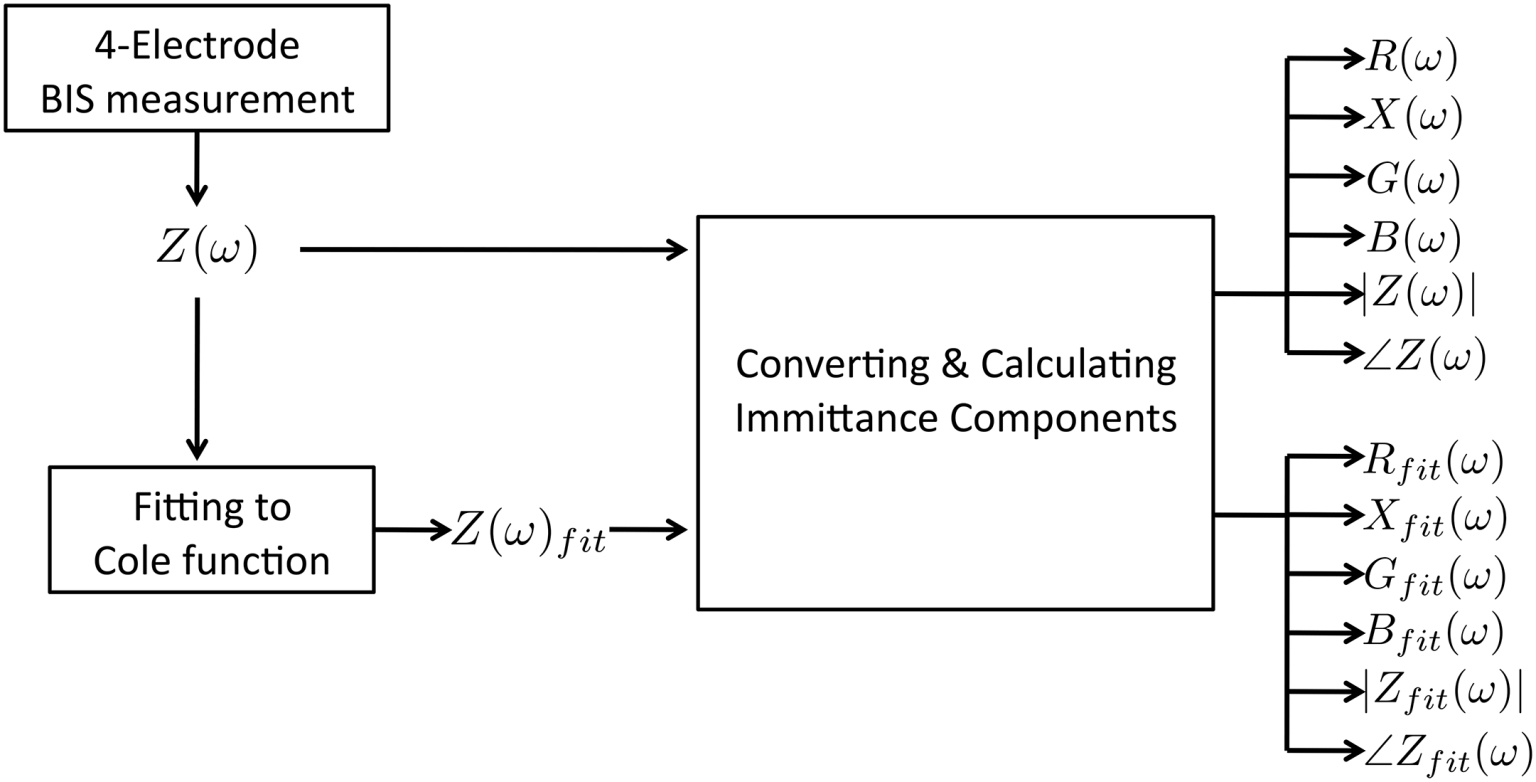
Steps to calculate the true and fitted spectral immittance components.

In this case of study, the experimental measurements produce complex impedance spectrums, which are fitted to Cole function with the corresponding parameters. After that, both admittance versions of the spectral impedance measurements and impedance Cole fittings are calculated, as shown in Fig 4. Once all the spectral immittance components are available, the features are calculated over the resistance, reactance, conductance, susceptance, impedance module |*Z*(*ω*)| and the impedance phase ∠*Z*(*ω*).

### 3.2 Proposed Features

Let us denote *Z*(*ω*) as a BIS measurement and *Z*_*fit*_(*ω*) as its estimated value using the Cole parameters obtained by fitting the measurement *Z*(*ω*) to the Cole model. The fitting method used in this work is the impedance plane fitting method proposed in [25]. The relative error between the BIS measurement *Z*(*ω*) and its estimated value *Z*_*fit*_(*ω*) is considered for the next six magnitudes: resistance (*R*(*ω*)), reactance (*X*(*ω*)), conductance (*G*(*ω*)), susceptance (*B*(*ω*)), impedance module (|*Z*(*ω*)|) and impedance angle (∠*Z*(*ω*)). In addition, those six errors are calculated in the five frequency bands defined in section 1.1. Since the frequency bands were defined with respect to the characteristic frequency, the relative errors eliminates any potential dependency between measurement application. Considering this, 30 features are proposed for classification: the mean relative errors of the six magnitudes split in five frequency bands. The 30 features are labeled according to Table 2, where <∗> represents mean value. An extra feature is included in the set, the sign of the reactance in the maximum frequency:

$$\begin{array}{c} f_{31}=sign\left(X\left(\omega_{max}\right)\right). \end{array}$$ (2)

**Table 2:** Proposed features for BIS measurement classification.

*f*_1_:	$<\frac{R\left(VLF\right)-R_{fit}\left(VLF\right)}{R(VLF)}>$	*f*_11_:	$<\frac{G\left(VLF\right)-G_{fit}\left(VLF\right)}{G(VLF)}>$	*f*_21_:	$<\frac{\left|Z\left(VLF\right)|-\right|Z_{fit}\left(VLF\right)|}{\left|Z(VLF)\right|}>$
*f*_2_:	$<\frac{R\left(LF\right)-R_{fit}\left(LF\right)}{R(LF)}>$	*f*_12_:	$<\frac{G\left(LF\right)-G_{fit}\left(LF\right)}{G(LF)}>$	*f*_22_:	$<\frac{\left|Z\left(LF\right)|-\right|Z_{fit}\left(LF\right)|}{\left|Z(LF)\right|}>$
*f*_3_:	$<\frac{R\left(MF\right)-R_{fit}\left(MF\right)}{R(MF)}>$	*f*_13_:	$<\frac{G\left(MF\right)-G_{fit}\left(MF\right)}{G(MF)}>$	*f*_23_:	$<\frac{\left|Z\left(MF\right)|-\right|Z_{fit}\left(MF\right)|}{\left|Z(MF)\right|}>$
*f*_4_:	$<\frac{R\left(HF\right)-R_{fit}\left(HF\right)}{R(HF)}>$	*f*_14_:	$<\frac{G\left(HF\right)-G_{fit}\left(HF\right)}{G(HF)}>$	*f*_24_:	$<\frac{\left|Z\left(HF\right)|-\right|Z_{fit}\left(HF\right)|}{\left|Z(HF)\right|}>$
*f*_5_:	$<\frac{R\left(VHF\right)-R_{fit}\left(VHF\right)}{R(VHF)}>$	*f*_15_:	$<\frac{G\left(VHF\right)-G_{fit}\left(VHF\right)}{G(VHF)}>$	*f*_25_:	$<\frac{\left|Z\left(VHF\right)|-\right|Z_{fit}\left(VHF\right)|}{\left|Z(VHF)\right|}>$
*f*_6_:	$<\frac{X\left(VLF\right)-X_{fit}\left(VLF\right)}{X(VLF)}>$	*f*_16_:	$<\frac{B\left(VLF\right)-B_{fit}\left(VLF\right)}{B(VLF)}>$	*f*_26_:	$<\frac{∠Z\left(VLF\right)-∠Z_{fit}\left(VLF\right)}{∠Z(VLF)}>$
*f*_7_:	$<\frac{X\left(LF\right)-X_{fit}\left(LF\right)}{X(LF)}>$	*f*_17_:	$<\frac{B\left(LF\right)-B_{fit}\left(LF\right)}{B(LF)}>$	*f*_27_:	$<\frac{∠Z\left(LF\right)-∠Z_{fit}\left(LF\right)}{∠Z(LF)}>$
*f*_8_:	$<\frac{X\left(MF\right)-X_{fit}\left(MF\right)}{X(MF)}>$	*f*_18_:	$<\frac{B\left(MF\right)-B_{fit}\left(MF\right)}{B(MF)}>$	*f*_28_:	$<\frac{∠Z\left(MF\right)-∠Z_{fit}\left(MF\right)}{∠Z(MF)}>$
*f*_9_:	$<\frac{X\left(HF\right)-X_{fit}\left(HF\right)}{X(HF)}>$	*f*_19_:	$<\frac{B\left(HF\right)-B_{fit}\left(HF\right)}{B(HF)}>$	*f*_29_:	$<\frac{∠Z\left(HF\right)-∠Z_{fit}\left(HF\right)}{∠Z(HF)}>$
*f*_10_:	$<\frac{X\left(VHF\right)-X_{fit}\left(VHF\right)}{X(VHF)}>$	*f*_20_:	$<\frac{B\left(VHF\right)-B_{fit}\left(VHF\right)}{B(VHF)}>$	*f*_30_:	$<\frac{∠Z\left(VHF\right)-∠Z_{fit}\left(VHF\right)}{∠Z(VHF)}>$

### 3.3 Least Squares Linear Discriminant Analysis

The linear discriminant analysis (LDA) is a supervised pattern recognition method that uses a linear combination of a set of input features in order to tackle a classification problem, establishing linear decision boundaries between two or more classes. Let us consider the pattern vector **x**_**i**_ (i.e. the observations) containing *P* input features, **x**_**i**_ = [*x*_1_, *x*_2_, …, *x*_*P*_]^*T*^. Each pattern **x**_**i**_ can be assigned to one of the *C* possible classes (i.e. error types in this work). The pattern matrix **P** of size *P*x*L* is defined as a matrix that contains the patterns **x**_**i**_ of a set of *L* data samples, **P** = [**x**_**1**_, …, **x**_**L**_ **]**, and the matrix **Q** is defined as

$$\begin{array}{c} Q=[\begin{array}{c} 1\\ P \end{array}], \end{array}$$ (3)

where 1 is a row vector of length *L*. In the case of multi-class LDA, the output is obtained computing *C* different linear combinations of the input features, according to

$$\begin{array}{c} Y=VQ, \end{array}$$ (4)

where V is the weight matrix that contains the bias *v*_0*c*_ and the weights *v*_*pc*_ applied to each of the *P* input features for each class *c*, and it is defined as:

$$\begin{array}{c} V=[\begin{array}{ccc} v_{10} & \cdots & v_{1P}\\ \vdots & \ddots & \vdots \\ v_{C0} & \cdots & v_{CP}. \end{array}] \end{array}$$ (5)

The LDA output **Y** is a *C*x*L* matrix that contains *C* outputs for each data sample:

$$\begin{array}{c} Y=[\begin{array}{ccc} y_{11} & \cdots & y_{1L}\\ \vdots & \ddots & \vdots \\ y_{C1} & \cdots & y_{CL} \end{array}]. \end{array}$$ (6)

The decision rule is given by

$$\begin{array}{c} {\hat{c}}_{l}=\underset{c}{\text{max}}\;\;y_{l}, \end{array}$$ (7)

where ${\hat{c}}_{l}$ is the estimated class for the *l*-th data sample and **y**_*l*_ the *l*-th column of Y.

The objective during design is to find the weight matrix **V** that minimizes the classification error. In supervised learning, the true values associated to each data sample are accessible, and they are used to train the classifier. These values are contained in the target matrix **T** defined as

$$\begin{array}{c} T=[\begin{array}{ccc} t_{11} & \cdots & t_{1L}\\ \vdots & \ddots & \vdots \\ t_{C1} & \cdots & t_{CL} \end{array}], \end{array}$$ (8)

having each column **t**_*l*_ a value of ‘1’ in the position of the true class and ‘0’ in the remaining positions. The estimation error is defined as the difference between the output values of the LDA Eq (4) and the true values

$$\begin{array}{c} E=Y-T=VQ-T, \end{array}$$ (9)

and the mean squared error (MSE)

$$\begin{array}{c} MSE=\frac{1}{L}{∥Y-T∥}^{2}=\frac{1}{L}{∥VQ-T∥}^{2}. \end{array}$$ (10)

The least squares solution (LS-LDA) [26] adjusts the weights to minimize the MSE. This is obtained by deriving the expression Eq (10) with respect to every weight of the linear combination, giving raise to the following expression:

$$\begin{array}{c} V=TQ^{T}{\left(QQ^{T}\right)}^{-1}. \end{array}$$ (11)

### 3.4 Feature selection algorithm

In section 3.2 a total of 31 features were proposed to detect and classify BIS measurement errors. This number of features can be reduced using a feature selection algorithm. The main reason to reduce the number of features is to avoid overfitting. A learning algorithm is said to overfit relative to a simpler one if it is more accurate in fitting known data but less accurate in predicting new data. Overfitting generally occurs when a model is excessively complex. Hence, using a lower number of features for classification reduces the possibilities of overfitting. In addition, the computational cost of the classifier directly depends on the number of features, so it can be reduced using a lower number of features. The objective of the feature selection algorithm is the selection of a determined number of features (*N*_*FEAT*_) among the whole set optimizing a fitness function. In this case, the objective is to minimize the classification error, i.e. the percentage of samples misclassified by the classifier. Considering the total number of features, to perform an exhaustive search is not affordable. Consequently, a heuristic search of the space of all possible feature subsets is performed. Among heuristic methods for feature selection, wrappers are usually applied when the selection process relies on evaluating a particular fitness function [27]. Some examples of wrapper methods are simulated annealing [28], particle swarm optimization [29] or evolutionary algorithms [30].

The solution proposed in this work is based on evolutionary algorithms (EA), which are iterative methods inspired in natural evolution laws [31]. An EA is commonly build by 3 functional blocks [32]: 1)generation of candidate solutions (CSs), 2) evaluation of a fitness function (FF), and 3) the evolution of the population. The definition of CS is problem specific and they are built by a set of components that can have binary, discrete or continuous values. The optimization of the FF as cost function is also problem specific. Selection, crossover and mutation are the operations responsible for the evolution of the population.

The steps of the feature selection algorithm used in this paper are the next:

1. An initial population of CSs is produced. The performance of the EA algorithm depends heavily on the size of the population. A large population could generate larger genetic diversity, increasing the search space but subsequently suffering from a slower convergence. A very small population would evaluate a limited search space, increasing the probability of converging into a local extreme. In this work, the initial population contains 50 CCs. Each candidate solution contains *P* = 31 (the total number of features) random binary values (0 or 1), that indicates whether a feature is selected or not.
2. The candidates of the population are validated to fulfill the constraint of total number of features. If a candidate solution exceeds the maximum number of features (*N*_*FEAT*_) by a determined number, the same number of random positions are decreased by one (avoiding negative values). The process iterates until the candidate solution fulfills the requirement.
3. The fitness function is evaluated for each candidate solution of the population. To reduce the probability of overfitting, *k*-fold cross-validation [33] is used to evaluate each candidate. The classification error obtained by the LS-LDA classifier using the subset of features contained in the candidate solution is evaluated for each fold. The *k* classification errors from the folds are then averaged and used as fitness function.
4. A selection process is performed, using the results of the evaluation of the fitness function as ranking. In this work, we use truncation selection, and consists in selecting a subpopulation of the best 10% candidate solutions that best fit the fitness function. These best candidates will survive to the next generation, and they are denominated ‘parents’.
5. Recombining the parents with a crossover operator to breed the new generation. The remaining 90% solutions of the new generation are generated by crossover of the parents. In this EA, the crossover operator implemented is uniform crossover. The probability that the crossover operator is applied to each individual (crossover probability) is 0.5. With this crossover schema, the offspring has approximately half of the elements from the first parent and the other half from the second parent.
6. The offspring is randomly changed or mutated to maintain diversity within the population and to inhibit premature convergence to local extreme. In this EA, mutations are only applied to the candidate solutions of the new population that are duplicated. The best solution is always excluded in the application o mutations. Mutations consist of changing the value of a random position of the repeated candidate solutions, until all individuals in the population are different.
7. The steps 2-6 are repeated until 100 generations are evaluated.

The solution with the features remaining in the last iteration is considered the best solution.

### 3.5 Classification approach

#### 3.5.1 All-at-once approach

In this first approach, seven different classes are defined, according to Table 3. A LS-LDA classifier is designed to classify all the seven classes at the same time, selecting the best subset of features among the proposed set (31 features). When a measurement is classified as Class 1, it is an error-free measurement. On the other hand, if the measurement is classified into any of the other 6 classes, a measurement error has been detected and also classified.

**Table 3:** Classes definition: all-at-once approach.

Class	Measurement type
Class 1	Error free
Class 2	Error Type-A
Class 3	Error Type-B
Class 4	Error Type-C
Class 5	Error Type-D
Class 6	Error Type-E
Class 7	Error Type-F

#### 3.5.2 Divide and conquer approach

In this approach, we divide the previous classification problem with seven classes into easier subproblems merging some classes that seem difficult to differentiate. Thus, Type-B and Type-C errors are considered a unique class, as well as Type-E and Type-F errors. The new classification problem has five classes, which are defined in Table 4. In a first step, a LS-LDA classifier is designed to differentiate between those five classes, selecting the best subset of features among the proposed set (31 features). In a second step, another two LS-LDA classifiers are designed to separate subclases: Type-B and Type-C errors from Class 3, and Type-E and Type-F errors from Class 5.

**Table 4:** Classes definition: divide and conquer approach.

Class	Measurement type
Class 1	Error free
Class 2	Error Type-A
Class 3	Error Type-B+Type-C
Class 4	Error Type-D
Class 5	Error Type-E+Type-F

In order to differentiate between subclasses of Class 3 and Class 5, we propose to use a binary LS-LDA that uses only one feature. Finding the appropriate feature makes possible to separate the subclasses. This is equivalent to establish a linear separation boundary or threshold between two classes (Type-B and Type-C or Type-E and Type-F). The criterium to select the feature is based on the Fisher score [34], which for two classes *c*_*i*_ and *c*_*j*_ is given by:

$$\begin{array}{c} F\left(f_{k}\right)=\frac{{\left(\mu_{k}^{ci}-\mu_{k}\right)}^{2}+{\left(\mu_{k}^{cj}-\mu_{k}\right)}^{2}}{\sigma_{k}^{2}}, \end{array}$$ (12)

where *F*(*f*_*k*_) is the Fisher score for the *k*-th feature, *μ*_*k*_ and *σ*_*k*_ denote the mean and standard deviation of the whole data set corresponding to the *k*-th feature, and $\mu_{k}^{ci}$ is the mean of the class *i*, corresponding to the *k*-th feature.

## 4 Experimental setup

A suitable database design plays a vital role in any kind of problem based on supervised machine learning. In order to design and validate the classification algorithms proposed in this work, a database of 1502 real BIS measurements has been created. The database is split into two different subsets, one for design and another for test. The design set contains the 60% of the measurements (901 samples), and the test set the remaining 40% (601 samples). Each class has the same number of samples in both sets, and the samples are randomly selected from the complete dataset. It is very important to emphasize that the test samples are not used in the design process.

**Fig 5:**
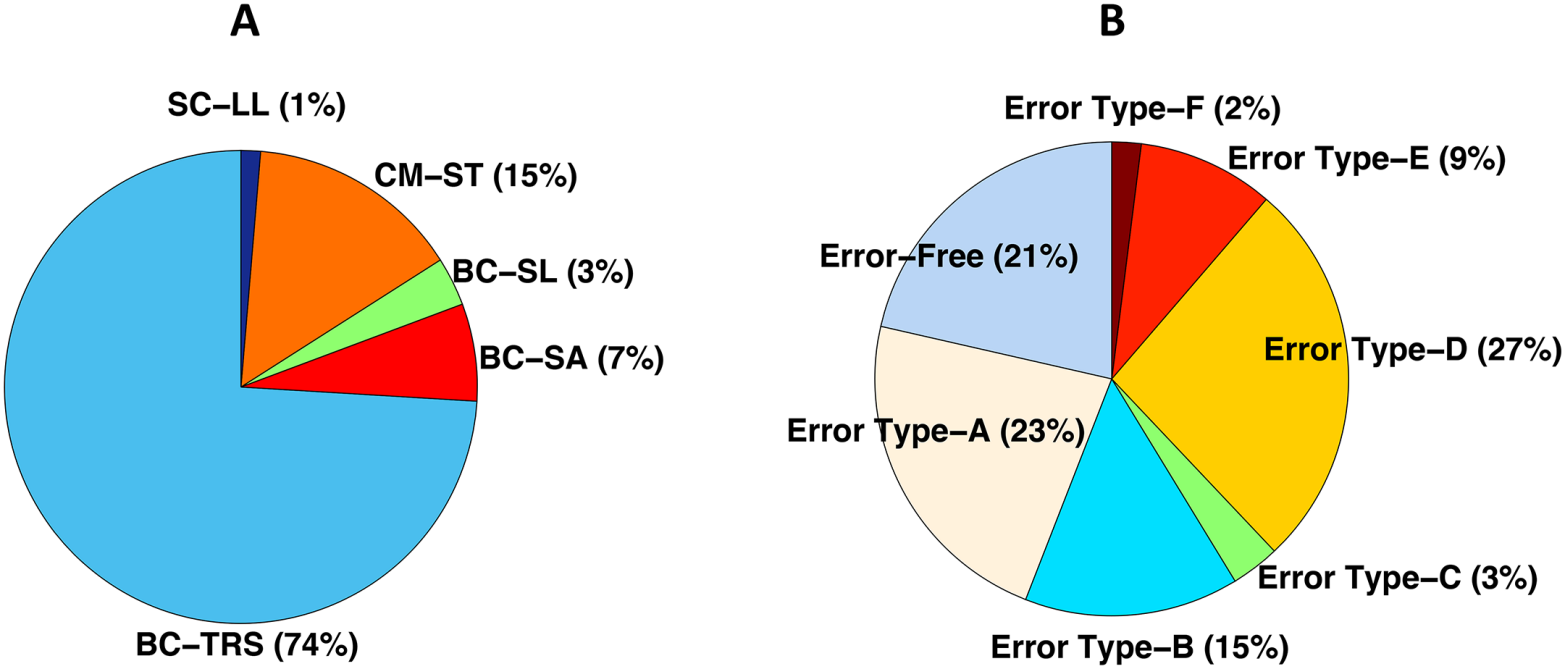
Distribution of the BIS experimental measurements included in the database. Distribution per application (A), distribution per measurement type (B).

The database contains 1502 complex spectra of BIS measurements obtained from different BIS applications: body composition with total right side (BC-TRS), segmental arm (BC-SA) and segmental leg (BC-SL) measurement types; cerebral monitoring with scalp transcephalic (CM-ST) measurement type; and skin-electrode interphase characterization with leg-to-leg (SC-LL) measurement type. The distribution of samples per application is shown in Fig 5(A). The database includes measurements with the six different types of error, explained in section 2, and error-free measurements. The number of samples for each measurement class is shown in Fig 5(B). The distribution of measurement errors and applications included in the database it is also very truthful with reality: not all the applications and types of errors happen with the same frequency in real life.

The study was approved by the Regional Ethical Review Board of Gothenburg (*Regionala etikprovningsnamnden i Goteborg*). Informed written consent to participate in the study was obtained from all the test subjects. The obtained measurements and the consent forms are kept according to the instructions given by the Regional Ethical Review Board of Gothenburg regarding the ethical approval for the study (Dnr 274-11).

## 5 Results

### 5.1 Results obtained with all-at-once approach

The feature selection algorithm is executed 31 different times, varying the maximum number of features (*N*_*FEAT*_) from 1 to 31 in steps of 1. Only the samples contained in the design set are used in this process. 5-fold cross-validation is used to evaluate the fitness function.

**Fig 6:**
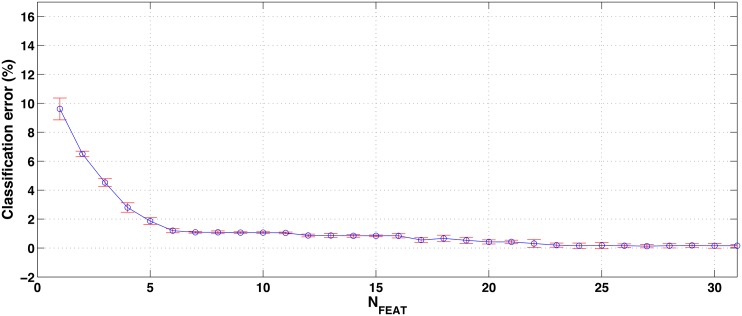
Mean and standard deviation (5 folds) of the classification error (%) obtained by the feature selection algorithm for different values of *N*_*FEAT*_ in the all-at-once approach (7 classes).

Fig 6 shows the mean and standard deviation of the classification error in the 5 folds, as a function of the number of features. Using all the 31 proposed features, the classification error is 0.16%, which corresponds with an average of 1.4 misclassified samples. The error is practically unaltered (even decreased) down to *N*_*FEAT*_ = 23, whereas the classification error begins to increase slightly for lower values. For a value of *N*_*FEAT*_ = 7, the classification error is still a 1.1% (an average of 1.8 misclassified samples), but for lower number of features the error starts to increase significantly. Additionally, the standard deviation of the classification error for *N*_*FEAT*_ = 7 is lower than in its neighbor points. According to this, we consider that *N*_*FEAT*_ = 7 represents a good tradeoff between classification performance and number of features (reduced overfitting probability and computational cost). The 7 selected features are shown in Table 5.

**Table 5:** Selected features with *N*_*FEAT*_ = 7 in the two classification approaches.

	Selected Features
**All-at-once**	*f*_5_, *f*_6_, *f*_7_, *f*_9_, *f*_18_, *f*_29_, *f*_31_
**Divide and conquer**	*f*_5_, *f*_7_, *f*_9_, *f*_26_, *f*_28_, *f*_29_, *f*_31_

Once the classifier has been designed, it is validated classifying the samples from the test set. The LS-LDA is trained with the samples of the design set using the 7 selected features. The classification error obtained by the designed classifier with the test samples is 5.7% (34 misclassified samples). In order to analyze the origin of those 34 misclassified samples, Table 6 shows the confusion matrix associated to the classifier. We can see that all the samples in Class 1, Class 3, Class 5 and Class 6 are perfectly classified. However, in the case of Class 2 (error Type-A), 2 samples are labeled as Class 5 (error Type-D), and in the case of Class 4 (error Type-C), 11 samples are labeled as Class 3 (error Type-B) and 9 samples are labeled as Class 5 (error Type-D). All the 12 samples in Class 7 are wrongly classified as Class 6 (i.e. error Type-F is misclassified as error Type-E).

**Table 6:** Confusion matrix in all-at-once approach. Classification error of 5.7%.

All-at-once	Class 1	Class 2	Class 3	Class 4	Class 5	Class 6	Class 7
Class 1	**129**	0	0	0	0	0	0
Class 2	0	**134**	0	0	2	0	0
Class 3	0	0	**88**	0	0	0	0
Class 4	0	0	11	**0**	9	0	0
Class 5	0	0	0	0	**160**	0	0
Class 6	0	0	0	0	0	**56**	0
Class 7	0	0	0	0	0	12	**0**

### 5.2 Results obtained with divide and conquer approach

In the current approach, the classification procedure is carried out in two steps. In the first step, a LS-LDA is trained to classify the 5 classes defined in Table 4. As in the previous approach, the feature selection algorithm is executed 31 different times, varying the maximum number of features (*N*_*FEAT*_) from 1 to 31. Only the samples contained in the design set are used in this process and 5-fold cross-validation is used to evaluate the fitness function.

**Fig 7:**
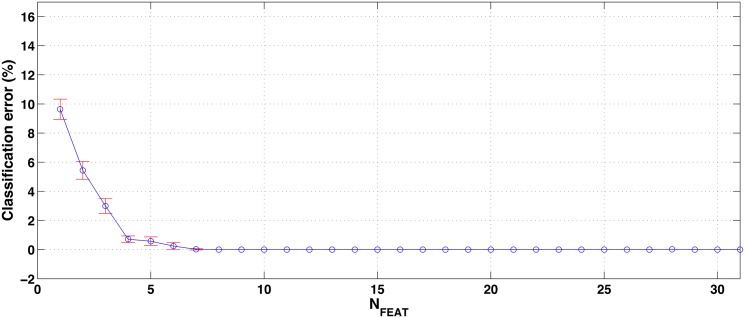
Mean and standard deviation (5 folds) of the classification error (%) obtained by the feature selection algorithm for different values of *N*_*FEAT*_ in the divide and conquer approach (5 classes).

The mean and standard deviation of the classification error, in the 5 folds, as a function of the number of features is represented in Fig 7. Using the maximum number of features (*N*_*FEAT*_ = 31) the classification error is 0%, and this value is kept down to *N*_*FEAT*_ = 8. In the case of *N*_*FEAT*_ = 7, the classification error is a 0.02% (an average of 0.2 samples misclassified) and the standard deviation is still very small. For lower number of features the mean and standard deviation of the classification error start to increase significantly. Similarly to the previous approach, we consider *N*_*FEAT*_ = 7 a good tradeoff between classification performance and number of features. The 7 selected features are shown in Table 5.

The designed classifier is validated with the samples from the test set. The LS-LDA is trained with the samples of the design set using the 7 selected features. The classification error obtained by the designed classifier with the test samples is 0.17% (1 misclassified sample). Table 7 shows the associated confusion matrix. Samples in Class 1, Class 3, Class 4 and Class 5 are perfectly classified, and only 1 sample of Class 2 is wrongly labeled as Class 4 (error Type-A labeled as error Type-D).

**Table 7:** Confusion matrix in divide and conquer approach, step 1. Classification error of 0.17%.

Divide and conquer-1	Class 1	Class 2	Class 3	Class 4	Class 5
Class 1	**129**	0	0	0	0
Class 2	0	**135**	0	1	0
Class 3	0	0	**108**	0	0
Class 4	0	0	0	**160**	0
Class 5	0	0	0	0	**68**

**Fig 8:**
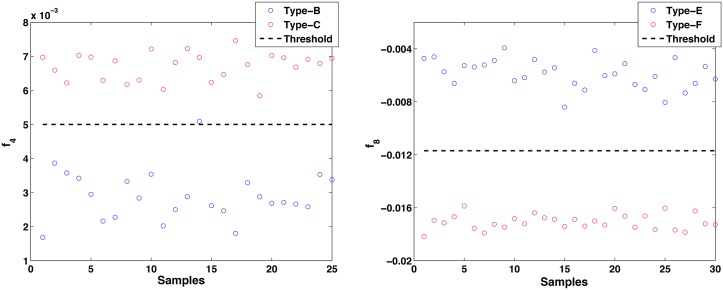
Feature *f*_4_ for samples of Type-B error and Type-C error in (A) and feature *f*_8_ for samples of Type-E error and Type-F error in (B). Samples are taken from different BIS applications.

Once the first step has classified the samples between the five classes, the second step sub-classifies samples labeled as Class 3 between Type-B or Type-C errors, and samples labeled as Class 5 between Type-E or Type-F errors. As it was previously proposed, subclasses are identified with a binary LS-LDA that uses only one feature selected by the Fisher Score criterium. Using the samples from the design set and according to Eq (12), the features selected are *f*_4_ in the case of Class 3 subclassification and *f*_8_ in the case of Class 5 subclassification. A binary LS-LDA is trained with Type-B and Type-C samples of *f*_4_ from the design set, using 5-fold cross-validation. The value of the threshold is obtained as the average of the thresholds obtained in each fold, $th_{BC}^{4}=0.005$. Fig 8 (left) represents the value of *f*_4_ for some samples of Type-B error (blue circles) and Type-C error (red squares), from different BIS applications. The threshold *th*_*BC*_ is represented by a black dashed line. The designed classifier then is evaluated with the samples of Type-B and Type-C errors from the test set. The result is that only 1 sample of Type-B error is misclassified as Type-C error, all samples of Type-C being correctly labeled. That means that a total of 1 sample of Class 3 is misclassified, which is equivalent to a classification error of 0.93%. The confusion matrix is shown in Table 8.

**Table 8:** Confusion matrix in divide and conquer approach, step 2 (BC). Classification error of 0.93%.

Class 3	Type B	Type C
Type B	**87**	1
Type C	0	**20**

Another binary LS-LDA is trained with Type-E and Type-F samples of *f*_8_ from the design set, using 5-fold cross-validation. The value of the threshold is obtained as the average of the thresholds obtained in each fold, $th_{EF}^{8}=-0.0117$. Fig 8 (right) represents the value of *f*_8_ for samples belonging to Type-E error (blue circles) and Type-F error (red squares), from different BIS applications, and the threshold *th*_*EF*_ (black dashed line). Finally, this third classifier is evaluated with the samples of Type-E and Type-F errors from the test set. The result is that all samples of both types of errors are correctly classified, the classification error being a 0%. The confusion matrix is shown in Table 9.

**Table 9:** Confusion matrix in divide and conquer approach, step 2 (EF). Classification error of 0%.

Class 5	Type E	Type F
Type E	**56**	0
Type F	0	**12**

On the whole, the divide and conquer approach with *N*_*FEAT*_ = 7 only misclassifies 2 samples from the test set: 1 sample in the first step and 1 sample in the second step (Class 3 subclasses). This is equivalent to a classification error of 0.33%. A summary of both classification approaches is shown in Table 10.

**Table 10:** Classification error of the two proposed approaches with *N*_*FEAT*_ = 7.

	All-at-once	Divide and conquer		
		Step 1	Step 2	Total
Classification Error	**5.7%**	0.17%	0.57%	**0.33%**
Misclassified samples	**34/601**	1/601	1/176	**2/601**

## 6 Discussion

In this study, a method to detect the presence of measurement artifacts and identify what kind of measurement error is present in a BIS measurement is proposed. The database used to design and validate the method contains measurements with the most common measurement errors reported in BIS. The database is not only heterogeneous from the error perspective but also from application perspective, including measurements from different applications. The distribution of errors and applications included in the database is also very truthful with reality, not all the applications and type of errors are represented in the same proportion. The most common errors and the most common applications contain more samples than the least common applications and the least common error types.

The fact that the number of samples of each class present in the database is different increases the probability of overfitting: the classifier can be overtrained to classify classes with a higher number of samples. In order to increase generalization, we have proposed the use of a simple classification schema as well as performed dimensionality reduction by means of a feature selection algorithm.

It is well known that different BIS applications have different typical immittance values. Defining features based on relative errors avoids the need for normalizing or standardizing the value of the features to eliminate the influence of differences in the magnitude of BIS measurements for different applications. Moreover, the use of the characteristic frequency when defining the frequency windows for calculating the relative errors eliminates any potential dependency between measurement application and the values of the calculated feature. Moreover, the fitting method used to estimate the reference impedance, which is used to calculate the relative error, is carried out in the complex impedance plane with the method proposed in [25]. This is motivated by the fact that measurement artefacts affect both the real and imaginary part of the impedance.

Obtaining a BIS measurement useful for data analysis is of paramount importance. Hence, the quality of the measurement and the presence of measurement artifacts is evaluated upon measurement acquisition. If possible, such detection should be done already by the spectrometer itself indicating if the measurement is free from artifacts or otherwise it contains certain measurement errors and which kind. In some cases, the measurement error might be corrected or compensated by software and, in other cases, the best option will be to repeat the measurement after assessing the good placement of electrodes and other sources for potential measurement errors. The use of a classification engine based on linear discriminants decreases the complexity allowing a simple implementation without requiring extensive computational resources from the measurement system.

The all-at-once classification approach obtained a relative low classification error of 5.7%. The most problematic classes were Class 4 and Class 7, all their samples being wrongly classified. In the case of Class 4, the larger number of misclassifications were as Class 3, and, in the case of Class 7, all the samples were labeled as Class 6. This is clearly due to the similarities between the true and the labeled measurement errors, which makes that a linear classifier is not able to separate perfectly the 7 classes at the same time. For that reason, the divide and conquer approach has been proposed. Merging the most problematic classes (i.e. the ones with more similarities) into one class and performing a two-step classification have decreased the classification error to 0.33%. This improvement is caused by the use of specific linear classifiers to differentiate between the most problematic classes, which turns easier the classification problem.

Regarding the selected features, it is interesting to observe that in both cases all the frequency ranges are represented among the 7 selected characteristics. It is remarkable but in a kind expected that the most common features between the two approaches belong to the two upper parts of the frequency spectrum: HF and VHF. Finally, it is also significant the absence of features obtained from the conductance and from the modulus of impedance. These two immittance magnitudes have shown certain robustness against capacitive leakage especially at low frequencies, therefore it is very likely that the conductance nor the modulus of the impedance will contain useful information about measurement errors.

## 7 Conclusions

Being able to ensure that a BIS measurement is free from measurement errors and it can be analyzed trusting the results is very important in clinical practice, since for people outside the field of BIS it is often extremely difficult to judge about the goodness of a BIS measurement. In this paper we have demonstrated that despite the different origins of measurement artifacts produced by parasitic stray capacitance, impedance mismatch, cross-talking or their very likely combination, it is possible to detect measurement errors present in a BIS measurement and identify which kind of artifact is present.

The possibility to identify what kind of measurement artifact is present in the BIS measurement is also of extreme importance for further analysis because in some cases and for some applications, certain measurement artifacts can be corrected, minimized or avoided. An accurate identification is useful even in the cases with measurement errors without available methods for correction or minimization. A positive identification can be used as indicator for problems with skin-electrode interface, cabling etc., and then the measurement can be repeated right away after replacing the electrodes or ensuring adequate cabling. The use of simple features and a simple linear discriminant core as classification engine allows the implementation of this pre-processing task in the current hardware of bioimpedance spectrometers.

## Supporting Information

S1 File Error free measurements. (CSV)

S2 File Error Type-A measurements. (CSV)

S3 File Error Type-B measurements. (CSV)

S4 File Error Type-C measurements. (CSV)

S5 File Error Type-D measurements. (CSV)

S6 File Error Type-E measurements. (CSV)

S7 File Error Type-F measurements. (CSV)

S8 File Measurement frequencies. (CSV)
